# Biofortification of Yeast with Selenomethionine in Corn Hydrolysate: Influence of Different Metabolic Pathways

**DOI:** 10.1007/s12011-025-04963-w

**Published:** 2026-01-07

**Authors:** Layna Amorim Mota, Rubens Perez Calegari, Alana Uchôa Pinto, Pietro Sica, Marcelo Pego Gomes, Antonio Sampaio Baptista, Valter Arthur

**Affiliations:** 1 Nuclear Energy Center in Agriculture, University of São Paulo, Av. Centenário, 303, Piracicaba, 13416-000 SP Brazil; 2 Department of Biological and Chemical Engineering, Aarhus University (BCE-AU), Gustav Wieds Vej 10D, Aarhus, 8000 Denmark; 3 University of São Paulo, ESALQ – Department of Food Science and Technology, Av. Pádua Dias, 11, Piracicaba, 13418-900 SP Brazil

**Keywords:** Selenoamino acids, Organic selenium, *Saccharomyces cerevisiae*, Corn hydrolysate

## Abstract

Yeast biomass, the main protein source in distillers dried grains with solubles (DDGS), can be enriched with selenium (Se), enhancing the nutritional and functional value of this coproduct. Because Se deficiency affects large populations worldwide, Se-enriched yeasts represent a practical supplementation strategy, particularly in the organic form selenomethionine (SeMet), which is highly bioavailable. This study investigated Se accumulation and speciation in *Saccharomyces cerevisiae* Thermosacc^®^ strain cultivated in corn hydrolysate under aerobic (AE0, AE200, AE400) and anaerobic (AN0, AN200, AN400) conditions, with Na₂SeO₃ concentrations of 0, 200, and 400 mg L⁻¹. Cell performance, total Se, organic Se, and SeMet were quantified. The highest Se accumulation occurred at 400 mg L⁻¹, while 200 mg L⁻¹ favored more efficient conversion into organic forms. Aerobic metabolism supported superior intracellular concentrations (total Se: 6.15 mg g⁻¹; organic Se: 3.47 mg g⁻¹; SeMet: 2.6 mg g⁻¹), exceeding values commonly reported for commercial Se-enriched yeasts (1–2 mg g⁻¹). Conversion efficiency into organic Se ranged from 53% to 79%, with intermediate Na₂SeO₃ supplementation yielding the most favorable balance between accumulation and transformation. These findings show that both Na₂SeO₃ dose and metabolic pathway strongly influence Se uptake and biotransformation. Cultivation in corn hydrolysate under aerobic conditions not only promoted higher SeMet formation but also reflects conditions relevant to industrial DDGS production. This approach provides a promising strategy to valorize an abundant ethanol coproduct into functional feed with improved selenium bioavailability and reduced risk of toxicity.

## Introduction

Access to foods rich in vitamins and minerals is limited by socioeconomic and geographical factors worldwide [1]. It is estimated that more than two billion people are affected by micronutrient-deficient diets, representing one of the main challenges to achieving the United Nations Sustainable Development Goal number 2 (Zero Hunger) by 2030 [2]. Among these micronutrients, selenium (Se) deserves special attention, as 0.5 to 1 billion people worldwide are at risk of insufficient intake of this trace element due to its uneven distribution in nature, which results in geographical variations in dietary availability [3–5]. Selenium is considered an essential biological trace element as well as an antioxidant micronutrient. The nutritional implications of Se depend on the chemical form in which it is available and the amount present in food [6].

During the growth of *Saccharomyces cerevisiae*, Na₂SeO₃, a potentially toxic and poorly bioavailable Se species, is converted into safer and highly bioactive forms with enhanced nutritional properties [7]. The majority of these bioactive species consist mainly of selenomethionine (SeMet), representing approximately 60–86% of the total, and selenocysteine (SeCys). Besides playing a crucial role in protein synthesis and antioxidant enzyme activity, these amino acids are also essential for several vital functions in living organisms, including antioxidant defense, inflammation reduction, thyroid hormone production, DNA synthesis, fertility, and reproduction [8]. Once incorporated into cells, SeMet can enter the sulfur (S) metabolic pathway, where it is converted into Se analogs of S-containing compounds [9, 10].

Se-enriched yeast can be used as a nutritional supplement, offering advantages such as the industrial-scale production of Se-enriched biomass, which is more practical compared to the use of other Se-accumulating organisms, such as plants, and represents a low-cost option [10]. In addition, yeast is an excellent source of proteins, lipids, and fibers, and its production with a high concentration of SeMet derived from inorganic Se is particularly advantageous [11, 12].

Co-products containing yeast, such as distillers dried grains with solubles (DDGS)—a by-product of the corn ethanol production process—have been widely employed as substitutes for soybean meal in animal feed. The use of DDGS is recognized for its nutritional and economic value, in addition to contributing to the sustainability of agro-industrial production [13]. Furthermore, Recent studies have explored novel technological approaches to enhance the value of DDGS [14].

The United States and Brazil, the two largest corn ethanol producers, generated 92.7 billion liters of ethanol and approximately 65 million tons of DDGS in the 2023/2024 harvest. In this context, the development of innovative analytical approaches aimed at synthesizing yeasts enriched with new compounds of interest, such as SeMet, is becoming increasingly relevant. These strategies can add value to DDGS, transforming it into a co-product of greater commercial relevance [15–17]. Moreover, maize is widely cultivated in worldwide, highlighting its versatility as a substrate for the production of biofortified yeasts (FAO 2025).

However, the ability of the microorganism to absorb Se and convert it into more bioactive molecules depends mainly on the cultivation conditions and the strain type. On average, the Se-enriched yeasts available commercially contain 1–2 mg Se g⁻¹ dry weight [19]. In this study, *Saccharomyces cerevisiae*Thermosacc^®^ strain, previously subjected to an evolutionary adaptation process in corn hydrolysate medium at high Na₂SeO₃ concentrations, was cultivated under both aerobic and anaerobic conditions. According to Lagunas (1979), *S. cerevisiae* is a facultative anaerobe and thus can develop through either anaerobic or aerobic metabolism. Among the differences between these two conditions, aerobic metabolism requires the presence of oxygen, which leads to complete oxidation and the production of 38 ATP per mole of glucose catabolized [20]. As a consequence, growth yield increases.

Based on this context, we formulated the following hypotheses:

1. Cell propagation under aerobic conditions favors the total accumulation of Se in *Saccharomyces cerevisiae* cells, resulting in higher intracellular concentrations of organic Se and SeMet.
2. The different metabolic pathways (aerobic and anaerobic) exhibit differences in the efficiency of converting inorganic Se into organic forms, leading to variations in the percentage of biotransformation.

The quantification of total Se in yeast, together with the bioactive portion—referred to as the organic Se fraction—present in yeasts adapted to high Na₂SeO₃ concentrations, was analyzed. It is important to emphasize that the mechanism of Se accumulation is not yet fully understood. Moreover, in contrast to previous investigations conducted in synthetic media, the present study employed corn hydrolysate, which more accurately represents industrial conditions. Therefore, the aim of this work was to investigate the accumulation and conversion of inorganic Se into organic Se and SeMet in yeasts cultivated in corn hydrolysate under aerobic and anaerobic conditions.

## Materials and methods

### Wort (Corn hydrolysate)

The wort was prepared in a continuously stirred tank reactor equipped with constant agitation and temperature control. Corn kernels were sourced from local suppliers in Piracicaba, Brazil, and ground into granules with a particle size of ≤ 2 mm. Prior to hydrolysis, the appropriate mass of ground corn and ultrapure water (MilliQ^®^) was measured and mixed at a ratio of 65 g of corn per 100 mL of water [21]. The pH of the mixture was then adjusted to 5.8 using 5 M NaOH.

The suspension was then heated to 87 ˚C, and α-amylase was added at a concentration of 0.1% (w/w) in a water-bath with temperature control, while the mixture was continuously agitated at 180 rpm for 150 min to induce starch gelatinization and dextrinization. Subsequently, the temperature was reduced to 65 ˚C, and amyloglucosidase was added at a concentration of 0.1% (w/w). The mixture was maintained at 65 ˚C under constant agitation (180 rpm) for an additional 150 min to complete the saccharification process, resulting in the final corn hydrolysate [22].

After the enzymatic hydrolysis, macro- and micronutrients were supplemented at the following rates: 10gL⁻¹ (NH₄)₂SO₄, 3gL⁻¹ KH₂PO₄ e 0.5gL⁻¹ MgSO₄, 4.5mgL⁻¹ ZnSO₄·7 H₂O, 0.3mgL⁻¹ CoCl₂·6 H₂O, 1mgL⁻¹ MnCl₂·4 H₂O, 0.1mgL⁻¹ CuSO₄·5 H₂O, 4.5mgL⁻¹ CaCl₂·2 H₂O, 3mgL⁻¹ FeSO₄·7 H₂O, 0.4mgL⁻¹ Na₂MoO₄·2 H₂O, 1mgL⁻¹ H₃BO₃, 0.1mgL⁻¹ KI, and 0.1mgL⁻¹ biotine.

### Yeast Adaptation

The *Saccharomyces cerevisiae* Thermosacc^®^ strain, a commercially available dehydrated active yeast commonly used in corn ethanol production, was sourced locally and stored dry without rehydration until the start of experimental trials. For Se biofortification, the yeast was gradually adapted to sodium selenite (Na₂SeO₃, 98% purity; Synth^®^, Brazil) through a stepwise exposure protocol. This adaptation involved incremental additions of Na₂SeO₃ (5 mg L⁻¹) to the corn hydrolysate medium, with an initial concentration of 240 mg L^− 1^ for 32 cycles until 400 mg L⁻¹, allowing the yeast to grow under progressively increasing concentrations of the compound, following the procedure described by Mota et al. [23].

### Cell Propagation Assays

A two-stage yeast propagation system was implemented under aerobic conditions using fed-batch fermentation in laboratory-scale stirred glass reactors. The setup was designed to evaluate the effects of sodium selenite (Na₂SeO₃) supplementation on yeast growth and selenium biofortification, using corn hydrolysate as the primary carbon source. Key process parameters including temperature, pH, aeration, and feeding rate were carefully controlled to simulate industrially relevant fermentation conditions. The detailed experimental procedures are presented in the subsections below.

### Aerobic Conditions

Yeast propagation under aerobic condition was carried out according to the treatments described in Table 1. Each treatment was conducted in five replicates using corn hydrolysate as the substrate, supplemented with the respective Na₂SeO₃ concentrations specified for each condition.

**Table 1:** Description of treatments for yeast cultivation under aerobic and anaerobic conditions

Treatment	Propagation metabolism	Concentration of Na₂SeO₃ (mg L⁻¹) added to the culture medium
AE0		0
AE200	Aerobiosis	200
AE400		400
AN0		0
AN200	Anaerobiosis	200
AN400		400

The experimental setup consisted of three laboratory-scale stirred glass reactors placed inside an incubator operating at 200 rpm, with temperature maintained at 30 ± 2 ˚C. Each reactor was connected to a computer-controlled peristaltic pump (Atlas Scientific, New York, USA) to regulate the feeding rate. Reactors were continuously stirred at 200 rpm, and temperature was maintained at 30 ± 2 ˚C.

For the aerobic condition treatments (AE), aeration was provided using compressed air (~ 21% O₂) delivered through a 2 μm diffusion stone (Humlegardens, Ekolager, Sweden) at a flow rate of 1 v v⁻¹ min⁻¹, ensuring a 1:1 ratio between aeration gas volume and reactor volume. This airflow was controlled via a mass flow controller (Brooks, Hatfield, USA). The substrate volume-to-aeration rate ratio remained constant throughout the process.

The yeast propagation process was divided into two stages. In the first stage, a preparatory step was performed to allow the yeast to adapt to the stress conditions of extremely low sugar concentrations (50–100 mg L⁻¹), facilitating a metabolic shift. This phase began with the addition of 7% (wet basis) of a previously adapted inoculum to 300 mL of corn hydrolysate, with an initial yeast concentration of 1.7 × 10⁷ cells mL⁻¹. The medium contained 5 g L⁻¹ of glucose and the corresponding concentration of Na₂SeO₃, as described in Table 1. This stage lasted 4 h, until total glucose depletion. Following this, the culture was transferred to a second 2 L continuously stirred tank reactor, for the second propagation stage.

In the second stage, the yeast culture was transferred to 300 mL of corn hydrolysate containing 100 mg L⁻¹ of glucose and adjusted to pH 5.8. Propagation was carried out in a fed-batch system with an initial feed rate of 0.208 mL min⁻¹. The feed rate was calculated as described by Lim and Shin [24], considering a specific growth rate of 0.155 h⁻¹ and a cell yield coefficient of 0.5 g of cells per g of sugar.

The second stage lasted 12 h, resulting in a total cultivation duration of 20 h. Subsequently, all material was centrifuged at 3000 *g* for 20 min at 4 ˚C and stored in a freezer (−80 ˚C) for later analyses.

### Anaerobic Conditions

The treatments evaluated in this stage were conducted in five replicates, using corn hydrolysate as the substrate supplemented with the respective Na₂SeO₃ concentrations specified for each treatment. Cultivation was performed in fed-batch mode until the total working volume of the bioreactor reached 2 L. Initially, 200 mL of wort containing 150 g L⁻¹ of glucose and adjusted to pH 5.8 was added to the reactor and inoculated with 8% (wet basis) of a previously adapted yeast inoculum. One hour after the start of the fermentation, feeding commenced using a peristaltic pump, delivering additional wort (150 g L⁻¹ glucose) at a flow rate of 1.66 mL min⁻¹ for 6.02 h, totaling 600 mL of hydrolysate.

Fermentation was conducted in three laboratory-scale stirred glass reactors, as previously described. The temperature was maintained at 30 ± 2 ˚C throughout the process. At the end of fermentation, the entire volume was centrifuged at 3,000 × g for 20 min at 4 ˚C, and the biomass was stored at − 80 ˚C for subsequent analyses.

### Analyses

#### Cellular Performance Parameters

Cell viability was determined by methylene blue staining according to the method described by Pierce [25]. The amount of biomass generated during the process was quantified based on dry weight, and the number of cells per mL was determined by direct counting using a Neubauer chamber, both as described by Zago et al. (1996). The biomass yield (Yx/s) was calculated according to Eq. 1 [27]:

1 $$\:{Y}_{\raisebox{1ex}{$x$}\!\left/\:\!\raisebox{-1ex}{$s$}\right.}=\:\frac{Xf-Xi}{Si-Sf}$$

Where *Xf* and *Xi* represent the final and initial biomass concentrations, respectively, while *Si* and *Sf* correspond to the initial and final substrate concentrations.

The specific growth rate (µ) was calculated based on the logarithmic variation in cell concentration over time (Eq. 2) [27].

2 $$\:\mu\:=\frac{{ln}Nf-{ln}Ni}{\varDelta\:t}$$

Where *Ni* and *Nf* represent the initial and final cell concentrations (cells mL⁻¹), respectively, and Δt corresponds to the time interval (in hours).

The ethanol content was determined by the distillation of 25 ml of sample followed by density measurement, as described by Calegari et al. [28].

#### Total Se Analysis

Selenium analyses were performed using inductively coupled plasma optical emission spectrometry (ICP-OES) on wort and yeast samples.

Before detection and quantification by ICP-OES, the samples were subjected to a digestion process with the addition of 6 mL of 20% nitric acid and 2 mL of 30% hydrogen peroxide in PTFE vessels using a microwave oven for 30 min, under 160 bar pressure and at 230 ˚C, according to the methodology described by Catarino et al. [29]. Subsequently, ultrapure water (resistivity 18.2 MΩ·cm) obtained from a Milli-Q water purification system was used for calibration curve construction. All chemicals used were of analytical reagent grade.

Plastic containers and glassware were cleaned by immersion in a 1.4 mol L⁻¹ nitric acid solution for 24 h. Subsequently, the containers and glassware were rinsed with ultrapure water, dried, and stored in a class 100 laminar flow cabinet. A stock standard solution of Se at a concentration of 1000 mg L⁻¹ was prepared and then diluted to construct the calibration curve.

After digestion, liquid samples were diluted 12.5-fold, while solid samples were diluted 100-fold. The ICP-OES was operated under the following conditions: radiofrequency generator power of 1.4 kW, frequency of 40 MHz, plasma gas flow of 1.5 L min⁻¹, auxiliary gas flow of 0.5 L min⁻¹, and carrier gas flow of 0.8 L min⁻¹. The solution uptake rate was maintained at 1.2 mL min⁻¹, with an integration time of 5 s. Selenium detection was carried out at a wavelength of 196.090 nm, according to the operating conditions described by Silva et al. [30].

#### Organic Se Analysis

The determination of organic selenium in the different treatments was preceded by the preparation of yeast samples. After the metabolic cultivation processes conducted under aerobic and anaerobic conditions, the aqueous yeast suspension was centrifuged (4500 *g*, 10 min, 4 ˚C), and the cellular pellet was washed three times with ultrapure water in order to remove medium residues and selenium not associated with the cells. Subsequently, the sediment containing the yeast cells was dried under vacuum until constant weight was achieved.

The dried yeast was then resuspended in ultrapure water and subjected to heating in a boiling water bath for 1 h to extract inorganic selenium. After this treatment, the samples were centrifuged again (8300 *g*, 15 min), and inorganic selenium was quantified in the supernatant [31]. This fraction was quantified by ICP-OES, according to the specifications described in Sect. 2.6.2 [32]. Considering that the total selenium content had been previously determined, the organic selenium content was calculated by difference, subtracting the inorganic fraction from the total selenium value.

#### SeMet Analysis

For SeMet analysis in the different treatments, yeast samples were initially washed with ultrapure water as described in item 2.6.3. Subsequently, cell biomass was disintegrated in a laboratory mill (Retsch^®^ MM 400) using tungsten beads of 0.3–0.5 mm at 4 ˚C. After disintegration, the obtained cell content was homogenized with ultrapure water using a vortex mixer.

The authentic SeMet standard (Table 2) and the yeast samples containing the analyte of interest were analyzed on an ACQUITY UPLC H-Class system (Waters Corporation) of ultra-performance liquid chromatography (UPLC) coupled to a diode array detector (DAD) and a Xevo TQ-S tandem quadrupole mass spectrometer (Waters Corporation, Milford, MA), operating with a Z-spray ionization source in positive mode. A volume of 5 µL of the authentic standard and the samples was injected into an Ascentis^®^ Express C8 column (100 × 4.6 mm; 2.7 μm particle size) from Supelco. The mobile phase consisted of 0.1% formic acid (solvent A) and methanol (solvent B). Analyte separation started with 10% B, then increased to 90% B within 5 min, held at 90% B for 1 min, and returned to the initial condition (10% B) within the following 6 min. The total run time was 12 min at a flow rate of 350 µL min⁻¹. The operating parameters of the Z-spray source were as follows: capillary voltage = 3.5 kV; source temperature = 150 ˚C; desolvation gas temperature = 300 ˚C; desolvation gas flow = 800 L h⁻¹.

The experiments for quantification of the SeMet analyte were carried out in Multiple Reaction Monitoring (MRM) mode, with conditions optimized as shown in Table 2.

**Table 2:** Precursor ion transitions, instrument settings, and retention time of the SeMet analyte

Compound	Precursor ion (m/z)	Product ion (m/z)	CV^a^ (V)	CE^b^ (eV)	*R*_T_^c^ (min)
**SeMet**	197,7*	180,6	45	10	3,50
	197,7**	56,1	18	15	3,50

^a^ CV – Cone Voltage

^b^ CE – Collision Energy

^c^ R_T_ - Retention Time of the analyte

* Quantification transition

** Confirmation transition

The analytical curve obtained in MRM mode was expressed in ng mL⁻¹. Reference standard solutions were prepared by appropriate dilutions of the stock solution of the selenomethionine analytical standard in H₂O (1.0 mg mL⁻¹), resulting in the following concentrations (ng mL⁻¹): 5, 10, 25, 50, and 100.

Yeast samples were weighed and dissolved in H₂O–methanol (50:50, v/v) containing 0.5% formic acid. Subsequently, the samples were vortexed for 5 min, centrifuged for 5 min at 6000 rpm, and filtered through a Millipore^®^ filter (0.45 μm). Triplicate injections were performed for both the standard solution and the samples. Data were acquired using MassLynx V.4.1 software (Waters Inc.) and processed with the TargetLynx™ Application Manager (Waters Corporation).

### Experimental Design and Statistical Analysis

The experiment followed a 2 × 3 factorial design, consisting of two metabolic conditions (aerobic and anaerobic) and three selenium doses (0, 200, and 400 mg L⁻¹), with five biological replicates per treatment, totaling 30 experimental units.

All response variables were tested using a two-way analysis of variance (ANOVA) to evaluate the main effects of metabolism (factor T), selenium dose (factor D), and their interaction (T × D). When significant effects were detected (*p* ≤ 0.05), treatment means were compared using Tukey’s post-hoc test. Differences among metabolism levels within each dose were denoted by uppercase letters, while differences among selenium doses within each metabolism were denoted by lowercase letters.

Descriptive statistics were expressed as mean ± standard deviation (SD), and the standard error of the mean (SEM) was calculated as SD/√n. All statistical analyses were performed in R software [33] (version 4.4.3) using the emmeans, multcomp, and tidyverse packages. Graphs and table formatting were performed in OriginPro 2022b (64-bit, SR1).

## Results and Discussion

### Cell Viability and Performance Parameters

The estimation of adequate Se levels in the diet requires information not only on the total Se content but also on the bioaccessible species present in the sample [34]. It has been reported that the biological efficacy of organic Se is approximately seven times higher than that of inorganic Se [35].

Mushtaq et al. [36] investigated the effects of various Se sources (Na₂SeO₃, SeMet, and Se-enriched yeast) on growth performance and antioxidant status of intensively reared silver carp. The Se source influenced growth performance, and the diet supplemented with 0.5 mg kg⁻¹ SeMet achieved the highest (*p* < 0.05) final body weight and specific growth rate compared to the other treatments. Similarly, alanine aminotransferase (ALT), aspartate aminotransferase (AST), and alkaline phosphatase (ALP) levels were also higher (*p* < 0.05) in the group supplemented with the same SeMet concentration.

Similarly, Muhammad et al. [37] reported the superior effect of Se in its organic form. They analyzed dietary supplementation of chickens with Na₂SeO₃, organic Se derived from yeast, and bacteria, evaluating egg yolk color, antioxidant profiles, and oxidative stability. The results showed that dietary Se supplementation significantly improved (*p* < 0.05) egg yolk color, the antioxidant profile of egg yolk and breast meat (total carotenoid and phenol content), with the organic Se treatment being superior in relation to the aforementioned parameters.

For the reasons outlined above, cell viability was evaluated throughout the propagation process under different metabolic conditions (aerobic and anaerobic), with varying Na₂SeO₃ concentrations, as well as cell performance parameters. In addition, total absorbed and adsorbed Se were quantified, along with the main form of organic Se accumulated within yeast cells previously adapted to high Na₂SeO₃ concentrations.

**Fig. 1:**
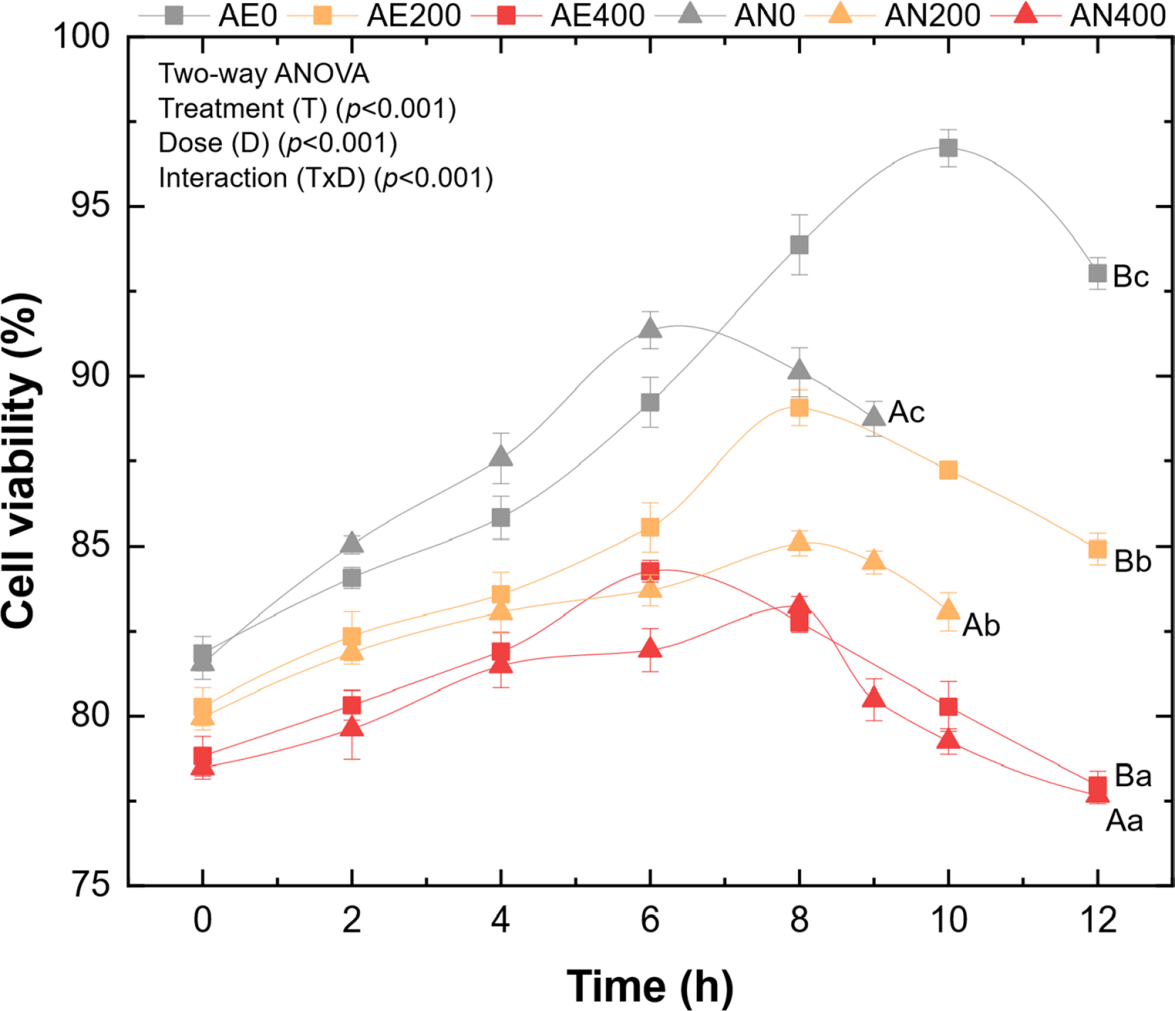
Cell viability of treatments under aerobic and anaerobic conditions, evaluated every two hours during the cultivation process. Values are expressed as mean ± SD (*n* = 5). Different uppercase letters indicate significant differences between metabolic conditions (AE vs. AN) within the same selenium dose. Different lowercase letters indicate significant differences among selenium doses within the same metabolic condition

According to Fig. 1, the addition of Na₂SeO₃ to the culture medium negatively affected cell viability in both aerobic and anaerobic treatments. This relationship proved to be inversely proportional, as increasing Na₂SeO₃ concentration in the medium led to a progressive reduction in cell viability. In Fig. 1(A), AE0 ended the cultivation with 93.03 ± 0.47% cell viability, a value 15.1% points higher than that observed in the treatment with the highest Na₂SeO₃ concentration (AE400: 77.96 ± 0.42%).

The two-way ANOVA revealed that metabolism (T), selenium dose (D), and their interaction (T×D) significantly affected the final cell viability (*p* < 0.001). Tukey’s test indicated that, within each metabolism, the three selenium doses belonged to distinct homogeneous groups, confirming a clear dose-dependent decline. Likewise, within each dose, aerobic treatments consistently showed higher viability than anaerobic ones (Table 3), demonstrating the strong influence of oxygen availability on Se tolerance.

**Table 3:** Effect of metabolic conditions on selenium accumulation, biomass production, and fermentation performance of S. cerevisiae

Item	Metabolism	Selenium dose				*p*-value		
		0 mg L⁻¹	200 mg L⁻¹	400 mg L⁻¹	SEM	T	D	T×D
Total Se (mg g^− 1^ DW)	AE	0.67 ± 0.10 Aa	2.83 ± 0.13 Bb	6.15 ± 0.20 Bc	0.059	< 0.001	< 0.001	0.060
	AN	0.50 ± 0.05 Aa	2.52 ± 0.13 Ab	5.67 ± 0.18 Ac				
Organic Se (mg kg⁻¹ DW)	AE	0.36 ± 0.06 Aa	2.22 ± 0.11 Bb	3.47 ± 0.11 Bc	0.036	< 0.001	< 0.001	< 0.001
	AN	0.26 ± 0.03 Aa	1.85 ± 0.09 Ab	3.03 ± 0.10 Ac				
SeMet (mg kg⁻¹ DW)	AE	0.22 ± 0.03 Aa	1.45 ± 0.07 Bb	2.61 ± 0.09 Bc	0.027	< 0.001	< 0.001	< 0.009
	AN	0.17 ± 0.02 Aa	1.27 ± 0.07 A6b	2.36 ± 0.08 Ac				
Biomass (g L⁻¹)	AE	41.27 ± 0.92 Bc	32.56 ± 0.76 Bb	24.79 ± 0.56 Ba	0.205	< 0.001	< 0.001	< 0.001
	AN	10.43 ± 0.10 Ab	9.34 ± 0.28 Aa	8.88 ± 0.13 Aa				
log₁₀ Cells (mL⁻¹)	AE	9.27 ± 0.04 Bc	8.94 ± 0.01 Bb	8.78 ± 0.02 Ba	0.010	< 0.001	< 0.001	< 0.001
	AN	8.57 ± 0.03 Ac	8.30 ± 0.03 Ab	8.31 ± 0.02 Aa				
µ (h⁻¹)	AE	0.16 ± 0.00 Bc	0.14 ± 0.00 Bb	0.12 ± 0.00 Ba	0.001	< 0.001	< 0.001	< 0.001
	AN	0.08 ± 0.00 Ac	0.07 ± 0.00 Ab	0.05 ± 0.00 Aa				
Yxs (g g⁻¹)	AE	0.52 ± 0.01 Bc	0.45 ± 0.01 Bb	0.21 ± 0.00 Aa	0.007	< 0.001	< 0.001	< 0.001
	AN	0.35 ± 0.03 Ac	0.32 ± 0.04 Ab	0.26 ± 0.01 Ba				
Final viability (%)	AE	93.03 ± 0.47 Bc	84.92 ± 0.46 Bb	77.96 ± 0.42 Aa	0.210	< 0.001	< 0.001	< 0.001
	AN	88.75 ± 0.52 Ac	83.07 ± 0.56 Ab	79.25 ± 0.38 Ba				
Final ethanol (%v v^− 1^)	AE	0.22 ± 0.02 Aa	0.51 ± 0.02 Ab	0.99 ± 0.03 Ac	0.030	< 0.001	0.023	< 0.001
	AN	8.02 ± 0.09Bc	7.77 ± 0.20 Bb	7.48 ± 0.05 Ba				

Values are expressed as mean ± SD (<i>n</i> = 5). Different uppercase letters indicate significant differences between metabolic conditions (AE vs. AN) within the same selenium dose. Different lowercase letters indicate significant differences among selenium doses within the same metabolic condition. T indicates the main effect of metabolic condition, D indicates the main effect of selenium dose, and T×D indicates their interaction. SEM stands for Standard Error of the Mean, calculated as SD/√n, and reflects the precision of the estimated mean. Statistical significance was set at <i>p</i> < 0.05. Cell counts were analyzed after log₁₀ transformation to meet the assumptions of ANOVA; the table reports the transformed means ± SD

The treatments conducted under anaerobic conditions (Fig. 1) indicated that AN0 had the highest cell viability at the end of the fermentative process, reaching 88.75 ± 0.52%, which was 9.5% points higher than AN400 (79.25 ± 0.38%). When comparing the control treatments under each metabolic condition (AE0 vs. AN0), AE0 completed propagation with a viability 4.3% points higher than that observed for AN0.

In addition to the reduction in cell viability, the anaerobic conditions revealed another important effect of Na₂SeO₃: the extension of cultivation duration. The specific growth rate (µ) values (Table 3) indicate that the presence of the compound prolonged the cell generation time, resulting in cultivation periods of 9 h (AN0), 10 h (AN200), and 12 h (AN400). Therefore, AN200 extended the cultivation time by 1 h, whereas AN400 doubled this increase, reaching a total of 12 h.

The reduction in cell viability, as well as the extension of cultivation time, was also reported by Martiniano et al. [38]. In that study, the authors produced six yeast strains, including *S. cerevisiae* 405, 174, and 193, enriched with 15 mg L⁻¹ Na₂SeO₃, using agroindustrial by-products as the culture medium. According to Kieliszek et al. [39], the slowdown in yeast growth may be associated with oxidative stress induced by the presence of Se in the culture medium, which can trigger lipid peroxidation processes—a type of oxidative damage caused by free radicals that affect the hydrophobic regions of cell membranes and ultimately disrupt the ionic homeostasis of the cell [40, 41].

These free radicals can be generated from Se through the direct oxidation of thiol groups present in proteins and in cysteine residues of reduced GSH. This process leads to the formation of intramolecular disulfide bonds, selenotrisulfides (S–Se–S), and selenosulfides (S–Se), thereby indirectly promoting the generation of reactive oxygen species (ROS) [42]. The ROS formed subsequently react with membrane lipids or their precursors, initiating a cascade of lipid peroxidation in which unsaturated fatty acids are oxidized to lipid radicals and lipid peroxyl radicals. These reactive intermediates further propagate oxidative damage in proteins and DNA, intensifying oxidative damage and leading to the formation of lipid hydroperoxides. The subsequent breakdown of these hydroperoxides gives rise to aldehydes, such as malondialdehyde (MDA), and hydrocarbons, such as ethane and ethylene, which are widely used as markers of lipid peroxidation [40].

It is possible to identify how Se negatively affected the performance parameters of the microorganism. Metabolism, selenium dose, and their interaction (two-way ANOVA) significantly affected all performance variables, including cell concentration, biomass production, µ, and Yx/s (*p* < 0.001). Tukey’s test confirmed distinct homogeneous groups within each metabolism and within each dose (Table 3), demonstrating a consistent, dose-dependent decline across parameters. In addition to lipid peroxidation, osmotic pressure arising from solutes present in the medium represents another factor capable of exerting a significant impact on microorganism performance. When exposed to a high-osmolarity environment, yeast cells undergo hyperosmotic shock, characterized by a rapid efflux of water, loss of turgor pressure, and cell shrinkage. During this process, water is transferred from the vacuole to the cytoplasm as an initial compensatory response to osmotic stress [43]. This pressure can inhibit cellular metabolism, compromise substrate utilization efficiency and consequently reducing process yields [44, 45].

Metabolism, selenium dose, and their interaction significantly affected all performance parameters (Table 3), including ethanol production, biomass, and Yx/s (*p* < 0.001). Under aerobic conditions, ethanol concentration increased progressively with selenium dose, and Tukey’s test confirmed significant differences among all treatments (AE0 = 0.22 ± 0.02%, AE200 = 0.51 ± 0.02%, AE400 = 0.99 ± 0.03%). This reflects the gradual redirection of carbon flux toward ethanol formation as Na₂SeO₃ increased, consistent with the inhibitory effects on biomass production and µ.

In contrast, ethanol concentrations were an order of magnitude higher under anaerobic conditions, ranging from 8.02 ± 0.09% (AN0) to 7.48 ± 0.05% (AN400). A statistical difference (*p* < 0.05) was observed for this treatment, demonstrating a dose-dependent reduction in ethanol even under fermentative metabolism. As expected, within each selenium dose, aerobic treatments produced significantly less ethanol than anaerobic ones, confirming the strong effect of oxygen availability on carbon partitioning.

This shift in carbon routing was directly reflected in biomass production and in the Yx/s values. Treatment AE0 exhibited the highest yield, reaching 0.52 ± 0.01 g g⁻¹, which is 48.6% higher than that obtained in the best-performing anaerobic treatment (AN0, 0.35 ± 0.03 g g⁻¹).All selenium doses formed distinct homogeneus groups within each metabolism (Tukey’s test), and aerobic treatments consistently outperformed anaerobic ones within each dose, confirming the combined effect of oxygen availability and selenium concentration on biomass yield. These results reinforce that the presence of oxygen, combined with adequate carbohydrate concentrations, enhances the metabolic efficiency of yeast for cellular growth, whereas the anaerobic environment directs the metabolic flux predominantly toward ethanol synthesis.

Under both metabolic conditions, the control treatment showed the highest µ, possibly due to the absence of Na₂SeO₃, with AE0 at 0.16 ± 0.00 h⁻¹, which was twice the value recorded for AN0 at 0.08 ± 0.00 h⁻¹. The two-way ANOVA confirmed that metabolism, selenium dose, and their interaction significantly affected µ (*p* < 0.001). Tukey’s test indicated that, within each metabolism, all selenium doses differed from one another (Table 3), demonstrating a clear dose-dependent reduction in µ. Moreover, within each dose, aerobic treatments exhibited significantly higher µ than anaerobic ones, highlighting the strong influence of oxygen availability on growth kinetics.

Based on literature, the prolongation of the generation time, which directly impacts µ, is attributed to the delay in the lag phase, as discussed by Kiesliszek et al. [46]. These researchers cultivated *Candida utilis* ATCC 9950 in 30 mg L⁻¹ Na₂SeO₃, and the duration of the lag phase was significantly longer (6 h) compared to the control medium (2 h). As a result of this extension, there was a marked decrease in the budding indices of the yeasts during the logarithmic phase. It is worth noting that the Na₂SeO₃ concentrations tested in the present study were 13 times higher than those reported by Kieliszek et al. [46], further emphasizing the strong selective pressure applied to the yeast cells.

Although the evolutionary adaptation process favored the development of yeast cells at high Na₂SeO₃ concentrations, such adaptation was not sufficient for the microorganisms to exhibit the same performance as the control treatments (Table 3). According to Herrero and Wellinger [47], yeast cells exposed to selenite are susceptible to the formation of DNA double-strand breaks (DSBs) and show elevated mutation rates. These breaks can be induced by ROS generated in the presence of selenite, which promote chemical alterations in DNA bases, compromising replication fork integrity and genomic stability in dividing cells. This may explain the reduction in performance observed.

To obtain safer products with higher Se content, it is necessary to develop yeast resources with greater conversion and accumulation capacity [48]. Bodnar et al. [49] described several benefits of producing and using Se-enriched yeast as a dietary supplement, which include containing mainly SeMet in its composition, being useful in countries with Se deficiency, enhancing plasma glutathione peroxidase (GPx) activity, enabling low-cost production of organic Se, and being the product most similar to Se in food form that is readily available for fortification or supplementation.

In Table 4, it can be observed that the Se concentrations added to the culture medium were effectively metabolized by yeast cells in all treatments. This observation is corroborated by the low amount of residual element in the wort after the cultivation process, indicating its consumption by the microorganism. The AN400 treatment showed the highest residual Se content in the wort, with 2.05 ± 0.12 mg L⁻¹, equivalent to 1.11% of the initial total Se concentration. Nevertheless, this value remains low, indicating that more than 98% of the supplemented salt was assimilated by the cells, meaning that more than 98% of the supplemented salt was assimilated.

**Table 4:** Quantification of total se in wort under aerobic and anaerobic conditions

Treatment	Total Se Concentration	
	Initial Wort (mg L^− 1^)	Final Wort (mg L^− 1^)
**AE0**	n.d.^a^	n.d.
**AE200**	90.34 ± 0.11	0.31 ± 0.07
**AE400**	185.55 ± 0.17	1.24 ± 0.06
**AN0**	n.d.	n.d.
**AN200**	91.03 ± 0.27	0.48 ± 0.06
**AN400**	184.63 ± 0.6	2.05 ± 0.12

^a^ Not Detected.

### Quantification of Different Se Fractions: Total, Organic, and SeMet (H1)

**Fig. 2:**
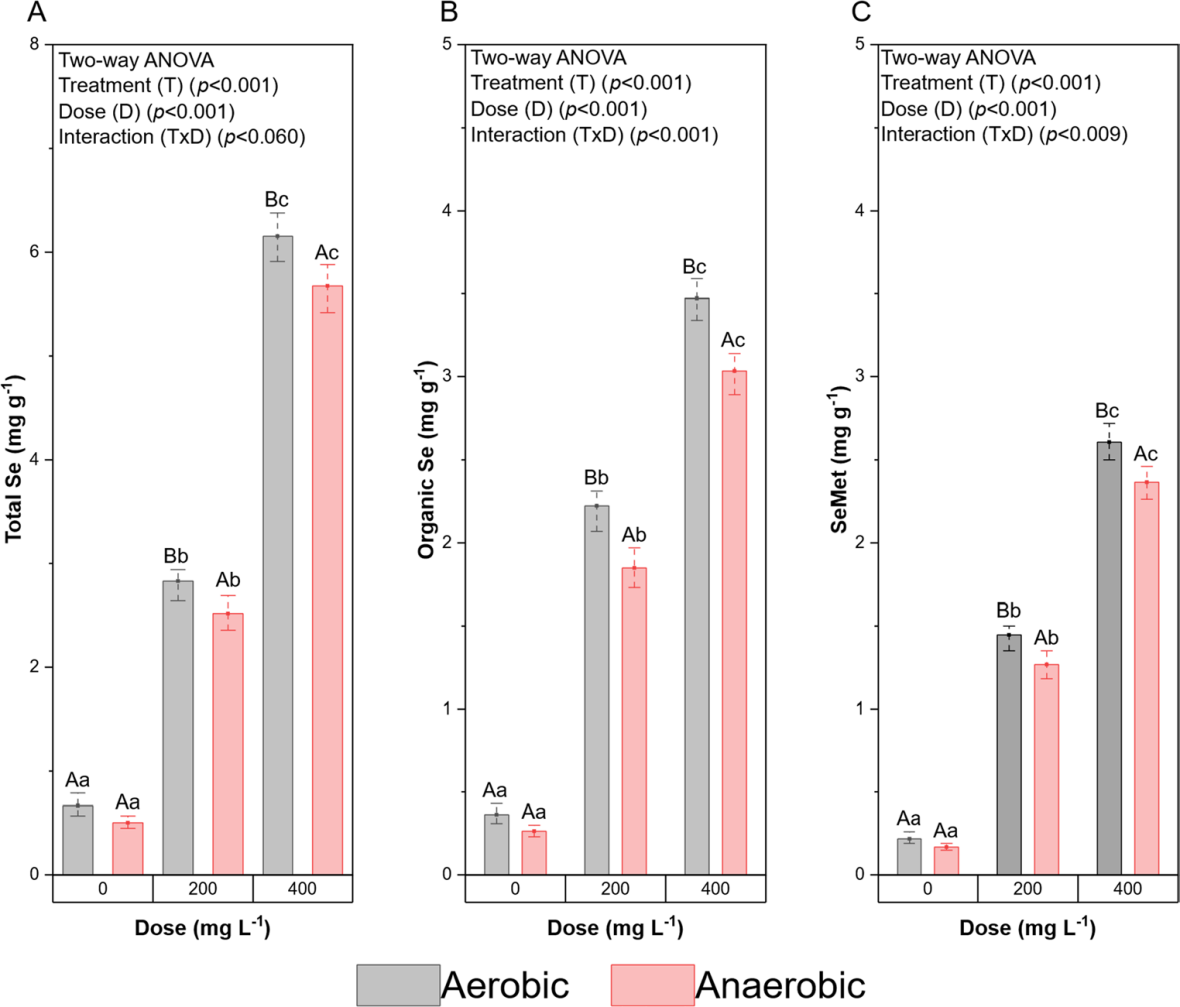
(**A**) Total Se quantification under aerobic conditions; (**B**) Total Se quantification under anaerobic conditions; (**C**) Organic Se quantification under aerobic conditions; (**D**) Organic Se quantification under anaerobic conditions; (**E**) SeMet quantification under aerobic conditions; and (**F**) SeMet quantification under anaerobic conditions. Results expressed on a dry weight basis

The first hypothesis tested in this study was that cell propagation under aerobic conditions favors the accumulation of total Se in *Saccharomyces cerevisiae* cells, resulting in higher intracellular concentrations of organic Se and SeMet. The results confirmed this premise, showing that under aerobic conditions there was greater accumulation of the different Se fractions analyzed compared to anaerobic conditions (Fig. 2).

Here, metabolism, selenium dose, and their interaction significantly affected total selenium, and SeMet accumulation (*p* < 0.001 for all treatments, two-way ANOVA). Tukey’s test showed that all selenium doses formed distinct homogeneous groups within each metabolic condition, confirming a clear dose-dependence accumulation pattern. Additionally, within each dose, aerobic treatments accumulated significantly more selenium than anaerobic ones.

Under aerobic conditions, treatment AE400 exhibited the highest total Se accumulation, reaching 6.15 ± 0.2 mg g⁻¹ of dry yeast, with significant differences among doses (*p* < 0.001). When this accumulation is compared with the other treatments within the same metabolic condition, AE400 accumulated approximately 2.2-fold more total Se than AE200 and 9.2-fold more than AE0, demonstrating a clear dose-dependent response to increasing Na₂SeO₃ concentrations.

Of the total Se quantified in AE400, 56.42% corresponded to the organic Se fraction (3.47 ± 0.11 mg g⁻¹) and 42.31% to SeMet (2.6 ± 0.09 mg g⁻¹), indicating substantial biotransformation capacity. Under anaerobic conditions, the AN400 treatment exhibited the highest accumulation, reaching 5.67 ± 0.18 mg g⁻¹ of total Se in dry yeast, also with significant differences among all treatments (*p* < 0.001). This value was 7.8% lower than that observed in AE400. Nevertheless, the maximum values obtained under both metabolic conditions exceeded those reported in previous studies, reinforcing the effectiveness of the adaptation and cultivation strategy employed.

Regarding organic Se and SeMet under anaerobic conditions, the highest accumulation was also observed in AN400. Of the 5.67 mg g⁻¹ of total Se, 53.44% corresponded to organic Se (3.03 ± 0.10 mg g⁻¹) and 41.62% to SeMet (2.36 ± 0.08 mg g⁻¹), percentages similar to those observed under aerobic conditions, although the absolute values were slightly lower. Two-way ANOVA showed significant effects of metabolism, selenium dose, and their interaction on both organic Se and SeMet accumulation (*p* < 0.001).

In the literature, no studies have yet reported the production of Se-enriched yeasts at high Na₂SeO₃ concentrations strictly under aerobic conditions to the best of our knowledge. However, this higher accumulation observed in the aerobic treatments can be explained in two ways: (1) Although propagation under aerobic conditions requires the presence of O₂, which favors the formation of free radicals and consequently induces oxidative stress, this condition also stimulates the activity of antioxidant enzymes to counteract the radicals formed [50]. In other words, this possible increase in enzyme activity may not be solely linked to the increment of Na₂SeO₃ in the culture medium but also to the mitigation of oxidative stress, thereby preserving cell function and ensuring better conditions for Se metabolization and accumulation. (2) The cells were previously subjected to an evolutionary adaptation process to high Na₂SeO₃ concentrations in the culture medium, which may have triggered adjustments in several metabolic pathways within the microorganism cells.

There are currently several Se-enriched yeast strains commercially available, such as Lalmin SE2000, Selenoexcell, and Sel-Plex. On average, these Se-enriched yeasts contain 1–2 mg Se g⁻¹ dry weight (Table 5). In the literature, some researchers have reported achieving more than 98% substitution of Met by SeMet and up to 3 mg total Se g⁻¹ dry weight [51, 52].

**Table 5:** Synthesis of studies reporting the quantification of total Se, organic Se, and SeMet in enriched yeasts

Reference	Species/strain	Cultivation condition	Total Se (mg g^− 1^)	Organic Se (% relative to total Se)	SeMet (% relative to total Se)
Present study	*Saccharomyces cerevisiae*/Thermosacc^®^	Aerobiosis in corn hydrolysate	0.67–6.15	53.73–78.8	32.83–58.53
Present study	*Saccharomyces cerevisiae*/Thermosacc^®^	Anaerobiosis in corn hydrolysate	0.5–5.67	52–73.41	34–50.39
Hachemi et al. [51]	*Saccharomyces cerevisiae* (comercial)/CNCM I-3060, CNCM I-3399, NCYC R397, NCYC R645 e NCYC R646	-	1–3	48.2–96.2	19–71.80%
Mapelli et al. [52]	*Saccharomyces cerevisiae/* CEN.PK113-7D (MATa, MAL2-8 C e SUC2) e CEN.PK113-5D (MATa ura3-52, MAL2-8 C e SUC2)	Aerobiosis in synthetic medium	≤ 2.6	≤ ~ 60	-
Kieliszek and Błazejak [53]	*Candida utilis/* ATCC 9950	Anaerobiosis in a medium composed of residual potato juice with 5% glycerol	≤ 0.63	**-**	~ 5%
Bierla et al. [54]	*Candida utilis* (Torula), commercial sample	-	3.6–4	**-**	7.3–11.6%
Bansal and Kaur [6]	*Saccharomyces cerevisiae*/MTCC Code 1766	Anaerobiosis in synthetic medium	≤ ~ 0,01	**-**	-
Esmaeili et al. [55]	*Saccharomyces cerevisiae*	Anaerobiosis in sugarcane molasses	0.1–0.3	86.44–96.45	-
Wang et al. [5]	*Pichia kudriavzevii*/1845	Aerobiosis	0.02–0.68	84.88	-
Rampler et al. [56]	*Saccharomyces cerevisiae/* Biomin Sey 38, BIO 19 e 696	Anaerobiosis with oxygen sparging in synthetic medium	1.3–4.2	-	70–100%

In the study conducted by Kieliszek and Błazejak [53], yeast biomass (*Candida utilis* ATCC 9950) was maintained in aqueous solutions enriched with inorganic Se (20 mg L⁻¹) for 24 h, and the total Se content accumulated was 629 µg g⁻¹ of yeast. In contrast, in the studies by Bierła et al. [54], total Se contents closer to those of the present study were reported, in which the yeast was able to accumulate 3.6 to 4 mg g⁻¹ dry weight, which is approximately twice the levels typically found in commercially available Se-enriched yeasts.

Under both metabolic conditions, it was observed that increasing Na₂SeO₃ concentration in the culture medium resulted in greater total Se accumulation, demonstrating its metabolization. Thus, the Na₂SeO₃ concentration added to the medium was directly proportional to the quantified total Se. Accordingly, the control treatments, under both aerobic and anaerobic conditions, showed the lowest total Se concentrations; nevertheless, AE0 (0.67 ± 0.1 mg g⁻¹) was 34% higher than AN0 (Fig. 2 (A), (B)).

In the study conducted by Bansal and Kaur [6], an increase in Se content in yeasts was also observed as the concentrations of added Se increased. They reported a significantly higher absorption of organic Se, which resulted in a substantial improvement in the total Se content of the cells.

In Hu et al. [57], the same behavior was observed in the biofortification of mushrooms with selenite, selenate, and SeMet. As a result, an increase in Se concentration in the fruiting bodies was reported, directly proportional to the increase of Se sources in the substrate. In addition, an increase in other trace elements was also identified.

Se can be accumulated in yeast cells in two ways: by absorption or by adsorption. Extracellular binding of Se occurs mainly through chemisorption, involving the formation of ionic interactions or the complexation of Se ions by biopolymers present in the yeast cell wall, such as active groups of proteins, phospholipids, or polysaccharides. The yeast cell wall is predominantly composed of polysaccharides and proteins, and its permeability may be influenced by the presence of long, branched carbohydrate chains that attach to the polypeptide chain via O-glycosidic bonds with hydroxyl groups of serine or threonine [58].

In general, Se biosorption occurs due to the presence of negatively charged functional groups on the cell wall surface, such as phosphodiester bridges, sulfides, and mannose phosphate residues [58–61].

With respect to intracellularly accumulated Se, or absorbed Se, some previous studies have noted that the process of intracellular Se accumulation occurs mainly through the active transport of other ions into yeast cells. To overcome the impermeability of the cell membrane to Se ions, the existence of a specific transport mechanism is essential. The known Se transport systems include the sulfate permeases SUL1 and SUL2 for selenate, the monocarboxylate transporter JEN1, as well as low-affinity (PHO87, PHO90, and PHO91) and high-affinity (PHO84 and PHO89) phosphate transporters for selenite [52, 62, 63].

The plasma membrane is the first cellular target involved in Se accumulation and transport regulation. 2,4-DNP (2,4-dinitrophenol) and CCCP (carbonyl cyanide m-chlorophenyl hydrazone) are uncouplers of oxidative phosphorylation and also cause rapid changes in membrane potential. KCN (potassium cyanide) and azide, on the other hand, interfere with cytochrome oxidase and act as respiratory inhibitors [64]. Nie et al. [48] described that, when Na₂SeO₄ enters the organism, the metabolic pathway is similar to that of thioamino acids, and the microorganism can detoxify excess selenate. The activated selenate is then reduced to selenite to synthesize SeMet and SeCys (considered the main organic forms of Se), noting that SeMet, with the help of cysteine, is a precursor for the production of SeCys.

Although yeast cells possess the ability to accumulate Se, this accumulation varies according to the cultivation conditions, particularly the presence of sulfate, sulfur-containing amino acids, glutamine in the medium, and the yeast strain type [65, 66]. In agreement with these observations, Asker et al. [67] evaluated the effect of Na₂SeO₃ concentration (from 0 to 1.04 g L⁻¹) and incubation periods (48 and 72 h) on Se bioaccumulation in yeast cells, including five *S. cerevisiae* strains and two *Candida* strains (*C. pseudotropicalis* and *C. tropicalis*). Among the yeast varieties, four showed the highest Se accumulation when cultivated with the highest Na₂SeO₃ concentration in the culture medium (1.04 g L⁻¹), whereas the *S. cerevisiae* MT3 strain exhibited greater accumulation when cultivated with 0.26 g L⁻¹ Na₂SeO₃, and *S. cerevisiae* MF with 3.0 mM Na₂SeO₃. With respect to the cultivation period, four of the examined varieties showed greater cellular Se accumulation after 72 h of cultivation, while the *S. cerevisiae* MF and MT3 strains demonstrated higher accumulation after 48 h of cultivation.

In the study conducted by Esmaeili et al. [55], the effects of cultivation conditions such as temperature, fermentation time, initial pH value, agitation speed, as well as the time and concentration of Se added to the medium, were evaluated on Se bioaccumulation in yeasts (*S. cerevisiae*). Fermentation was carried out under different temperatures (28–30 ˚C), initial pH values (4.5–5.8), agitation speeds (130–160 rpm), fermentation times (24–48 h), inoculum sizes (30–60 g L⁻¹), Se concentrations (15–25 µg mL⁻¹), and Se addition times (0–9 h). The results showed total Se accumulation and organic Se formation ranging from 107.9 to 287.6 mg kg⁻¹ and 93.27 to 269.05 mg kg⁻¹, respectively.

In Zhang et al. [68], batch cultivation of *Candida utilis* CCTCC M 209,298 under different pH conditions was analyzed for the preparation of Se-enriched yeast, with the aim of elucidating the physiological mechanism underlying the improved performance of Se-enriched yeasts under acid stress. RNA-Seq data indicated that 882 genes were significantly upregulated by moderate acid stress. These genes are involved in ATP synthesis and sulfur metabolism, including the biosynthesis of methionine, cysteine, and GSH in yeast cells. The increased intracellular supply of ATP and higher amounts of sulfur-containing compounds, in turn, contributed to the assimilation and biotransformation of Na₂SeO₃ into organic Se.

Another factor that may have influenced Se accumulation in the present study was the culture medium used, corn hydrolysate. Although all culture media are excellent sources of carbohydrates for metabolization, they present different nutrient concentrations [69], which can impact fermentation, propagation, and nutrient absorption processes. To date, no studies have been found in the literature that performed adaptive processes for Se accumulation and aerobic metabolism in microorganism propagation using corn hydrolysate.

The factors that determine Se accumulation or resistance to selenite may be influenced by selenite uptake or reduction rates, adsorption to the cell wall, and toxicity. All these factors are important to varying degrees in determining Se accumulation or resistance and are modulated by the medium [19].

According to the results, the levels of SeMet and organic Se in mg g⁻¹ dry yeast weight, under both aerobic and anaerobic conditions, showed significant differences (two-way ANOVA, *p* < 0.001 for metabolism, selenium dose, and T×D interaction; Fig. 2C–F).

When comparing the quantification of organic Se between the two metabolic conditions, it becomes evident that the treatments conducted under aerobic conditions consistently exhibited higher values than their anaerobic counterparts. AE0 (0.36 ± 0.06 mg g⁻¹) was 27.77% higher than AN0 (0.26 ± 0.03 mg g⁻¹). Similarly, AE200 (2.22 ± 0.11 mg g⁻¹) showed a value 17.04% higher than AN200 (1.85 ± 0.09 mg g⁻¹), whereas AE400 (3.47 ± 0.11 mg g⁻¹) was 12.69% higher than AN400 (3.03 ± 0.10 mg g⁻¹). These findings demonstrate that, regardless of the Na₂SeO₃ concentration, cells grown under aerobic conditions exhibited a greater capacity to convert inorganic Se into organic compounds.

The same pattern was observed for SeMet. AE0 (0.22 ± 0.03 mg g⁻¹) exhibited a value 22.72% higher than AN0 (0.17 ± 0.02 mg g⁻¹), whereas AE200 (1.45 ± 0.07 mg g⁻¹) was 11.80% higher than AN200 (1.27 ± 0.06 mg g⁻¹). Finally, AE400 (2.61 ± 0.09 mg g⁻¹) showed a value 9.23% higher than AN400 (2.36 ± 0.08 mg g⁻¹). Thus, for both organic Se and SeMet, the aerobic metabolism consistently demonstrated superior performance, reinforcing the crucial role of oxygen in the biotransformation of selenite into metabolically relevant organic forms.

It can be observed that treatments with higher Na₂SeO₃ concentrations (400 mg L⁻¹) exhibited greater accumulation of SeMet and organic Se. The organic Se fraction ranged from 52.08% to 78.64%, while SeMet ranged from 32.83% to 51.03% relative to the quantified total Se. In Hachemi et al. [51], a wide variation in organic Se quantification was reported; they analyzed several commercial Se-enriched yeasts and found values ranging from 48.2% to 96.2% organic Se relative to total Se. The range obtained in the present study falls within this interval, even when using an agro-industrial medium such as corn hydrolysate and conducting cultures under both aerobic and anaerobic metabolism. This indicates that the biotransformation efficiency achieved is comparable to, or even superior to, that of some commercial products.

In a more recent study, Wang et al. [5] analyzed the distribution and Se species in Se-enriched *P. kudriavzevii*. The results showed that *P. kudriavzevii* can accumulate Se and convert 84.88% into organic forms. Although our maximum values of organic Se (up to 78.64%) are slightly lower than those reported for *P. kudriavzevii*, they remain significant considering that *S. cerevisiae* exhibits distinct metabolic behavior and that our cultures were exposed to high concentrations of Na₂SeO₃ in a non-synthetic substrate.

Gharieb and Gadd [64] reported that SeMet was identified as the main Se compound present in the protein fraction and in whole yeast cells. Studies report that high-Se yeasts are predominantly composed of SeMet (74.8%), nonspecifically incorporated into peptide chains and highly digestible, followed by SeCys (9.9%), selenite (5.1%), and unknown Se compounds (10.2%). In agreement, Fagan et al. [34] mention that SeMet is the predominant compound found in Se-enriched yeast samples after aqueous extraction. The same pattern is also documented by Rampler et al. [56], where the production of Se-enriched yeasts using different *S. cerevisiae* strains showed around 70–100% SeMet, relative to the organic fraction of the product.

Contrary to this information, Nasim et al. [70] stated that although yeast can synthesize SeMet through the sulfur pathway, this is not the Se compound preferentially produced by *S. cerevisiae*. Therefore, the perception that Se-enriched yeast consists mainly of SeMet is not entirely accurate.

In line with this, Rao et al. [71] investigated the response of *S. cerevisiae* in accumulating Se when cultivated with different sources: SeMet, Na₂SeO₃, and Na₂SeO₄·10 H₂O. Among the various Se compounds found in *S. cerevisiae*, the rather unusual selenohomocysteine (Se-HCys) dominates, accounting for about 70% of the total Se content in the yeast. In contrast, the individual percentages of other Se compounds are below 10%. EFSA [72] reports that, overall, these yeasts contain up to 36% of total Se in the form of unspecified Se compounds. While Wu et al. [73], further demonstrated that the organic Se content in *S. cerevisiae* ranges from 1 to 4.5 mg g⁻¹ dry weight, and 58% of Se compounds are present as methylselenocysteine (MeSeCys).

In the study conducted by Hachemi et al. [51], the objective was to perform speciation of 13 types of Se-enriched commercial yeasts from different suppliers and batches, assessing total Se, inorganic Se species (SeIV, SeVI, and Se⁰), and organic Se species (SeMet and SeCys). The results showed that the proportion of Se as SeMet ranged from 19.0% to 71.8% (average of 55.8%), and a high variability in SeMet content was observed.

The yeast species is a determining factor for the type of Se predominantly accumulated intracellularly. Torula yeast (*Candida utilis*) was found to metabolize Se in a completely different way from *S. cerevisiae*, leading to the biosynthesis of selenohomolanthionine (SeHLan), an important Se compound accounting for 60–80% of total Se [54].

Although it is generally accepted that Se-containing compounds, peptides, and proteins in commercial Se-enriched yeasts are responsible for a range of health benefits, it was also noted that these products vary not only in their organoselenium metabolite profiles but also in their quantities [74].

Within this approach, the detection of selenocompounds in Se-enriched yeast products is of utmost importance, not only as a means of understanding the potential of the product but also to elucidate the fate of Se within the cell, thereby enabling its reproduction and ensuring product reliability.

The wide variation found in the literature regarding the proportion of SeMet formed is influenced by several variables, including the yeast species, the type of microorganism, the Se source, cultivation conditions, the type of wort, and additional external factors. SeMet is well known for its ability to be incorporated into proteins and antioxidant enzymes; however, it also acts as an antioxidant for small molecules [75].

Numerous organisms are unable to distinguish between SeMet and Met, resulting in a tendency to accept SeMet in various physiological processes as a substitute for Met. This phenomenon has significant consequences, since SeMet, being more reactive than Met, often acts as a redox cycler and catalyst [70]. For example, in Briviba et al. [76], SeMet and SeCys compounds protected dihydrorhodamine 123 from oxidation and 4-hydroxyphenylacetate from nitration more effectively than their sulfur analogs. In addition, it has been reported that Se-containing compounds are effective in protecting against peroxynitrite-induced single-strand DNA breaks.

The information presented highlights how the biotransformation of inorganic Se into organic Se by yeast can be beneficial. Additionally, the literature reports different applications of this product. For instance, in sheep, supplementation with Se-enriched yeast has been associated with increases in ruminal bacterial populations [77], while in goats it has been linked to improvements in testicular morphology and antioxidant status [78]. Studies indicate that organic sources of Se exhibit higher bioavailability, lower toxicity, reduced environmental impact, and greater assimilation efficiency compared to inorganic forms. In the specific case of SeMet, its structural similarity to Met facilitates intestinal absorption and incorporation into tissue proteins, forming an endogenous selenium reserve that can be mobilized during periods of stress to sustain additional selenoprotein synthesis [79]. SeMet may also act as a precursor for the formation of SeCys. According to Yu et al. [80], SeCys is incorporated into the catalytic center of iodothyronine deiodinases (DIO), enzymes that are critical for animal development. In addition, Se supplementation has been associate with increased digestive enzyme activities, directly contributing to improved animal growth.

### Se Conversion Rate (H2)

The second hypothesis tested in this study was that different metabolic pathways (aerobic and anaerobic) exhibit distinct efficiencies in converting inorganic Se into organic forms, resulting in variations in the percentage of biotransformation. The results confirmed this hypothesis, as the aerobic treatments showed higher conversion: AE0 was 1.73% higher than AN0, AE200 was 5.38% above AN200, and AE400 was 2.99% higher than AN400 (Fig. 2).

Also, it can be observed that the AE200 and AN200 treatments, with 200 mg L⁻¹ Na₂SeO₃ in the culture medium, showed the highest conversion rates of inorganic Se into organic Se, reaching 78.64% and 73.24% conversion, respectively. In contrast, the AE400 and AN400 treatments, which received the highest Na₂SeO₃ concentration, demonstrated conversions of 56.42% and 53.43%, respectively. Thus, AE200 and AN200 treatments exhibited on average 20% higher conversion to organic Se compared to the treatments with the greatest salt addition, highlighting the influence of Na₂SeO₃ concentration on the efficiency of Se metabolization by yeast.

A possible explanation for the higher conversion rate of inorganic Se into organic Se observed in the 200 mg L⁻¹ treatments (AE200 and AN200) is that, considering SeMet as the main form of intracellular Se storage, its storage capacity may have been reached, thus requiring the cells to activate alternative pathways to export or expel the excess Se. It should be noted that Se binds to methionine (Met) at the position normally occupied by S; therefore, as SeMet is formed, the concentration of Met decreases. However, Ouerdane and Mester [81] demonstrated that a specific yeast strain, BY4741, which is unable to synthesize Met using inorganic sulfur, produces SeMet in the presence of inorganic Se. This finding may indicate that the incorporation of inorganic Se salts does not occur exclusively through the same biological pathways as sulfur.

In Rampler et al. [56], reports can be found on the Met/SeMet ratio. They produced Se-enriched yeasts and monitored the fermentation process for 72 h under different Na₂SeO₃ feeding conditions. During fermentation, the Met/SeMet ratio continuously decreased, and at 47 h of fermentation a Met/SeMet ratio < 1.5 was reached in all strains. This fermentation point was considered critical for the yeast strains, as cell viability began to decrease by 2 to 10%.

A decrease in the proportion of SeMet could be explained by the transformation of the Se oxyanion into Se⁰, rather than into SeMet, during the cultivation process [51]. When yeast cells are exposed to high levels of selenite or selenate, they convert them into Se⁰ in an attempt to reduce their toxicity. It is well known that many microorganisms are capable of transforming the highly toxic, soluble oxyanion into a much less harmful, insoluble form, Se⁰ [82, 83].

Previous studies with *S. cerevisiae* indicated that resistance to SeMet may be associated with mutations in genes regulating sulfur amino acid metabolism [84]. Kitajima et al. [85] suggested that SeMet toxicity is due to Se compounds derived from SeMet, rather than an increase in the intracellular Met pool induced by blockage of the sulfur metabolic pathway or by the substitution of SeMet for Met residues in endogenous proteins.

Se accumulation in the cell depends on several factors, as already mentioned, and the same applies when comparing cultures under different metabolic conditions. In Wiebe et al. [86], for example, *S. cerevisiae* CEN.PK113-1 A was cultivated in a glucose-limited chemostat culture with 0%, 0.5%, 1.0%, 2.8%, and 20.9% O₂ in the inlet gas (D = 0.10 h⁻¹, pH 5, 30 ˚C) to determine the effects of oxygen on metabolite generation. It was observed that tricarboxylic acid (TCA) cycle metabolite concentrations were higher under anaerobic conditions, while 2-phosphoglycerate + 3-phosphoglycerate and phosphoenolpyruvate were higher under aerobic conditions.

Despite the promising results obtained in the present study, the efficiency of Se biotransformation will ultimately depend on the yeast strain and the metabolic pathways activated under different cultivation conditions. Thus, future research should focus on the development and refinement of strains with higher tolerance to elevated Na₂SeO₃ concentrations and with enhanced metabolic routing toward organic Se formation. This may include targeted manipulation of sulfur assimilation pathways, increased expression of genes involved in redox homeostasis (e.g., glutathione- and thioredoxin-dependent systems), or adaptive evolution strategies to improve robustness under industrial conditions.

Additionally, comparative omics analyses (transcriptomics, metabolomics, and proteomics) may elucidate specific metabolic bottlenecks that limit Se conversion, enabling the development of strains with superior bioconversion capacity. Such approaches would provide further insights for advancing the production of Se-enriched DDGS and strengthening the applicability of this coproduct in large-scale industrial processes.

### Relevance and Applicability in Industry

The present study introduces as an innovation the investigation of different metabolic processes using the same medium (corn hydrolysate) and inoculum (*Saccharomyces cerevisiae* Thermosacc strain) employed by the corn ethanol industry—an aspect not previously explored in the literature. This approach demonstrated the feasibility of generating higher value-added coproducts enriched with nutrients of high bioavailability and lower toxicity.

The advantages and applicability of DDGS enriched with high levels of organic Se, including SeMet, are associated with its greater bioavailability, potential for tissue storage as a reserve form, and reduced risk of toxicity. In addition, its inclusion in animal diets promotes the strengthening of the antioxidant system, enhances immune response, increases reproductive efficiency, and improves the quality of animal-derived products such as meat, milk, and eggs, which in turn exhibit higher selenium concentrations of nutritional relevance for human consumption [87, 88].

Studies reporting the promising use of selenium-enriched yeast in animal nutrition can be found in the literature. For example, Zhang et al. [89], investigated the effects of dietary supplementation with Se-enriched yeast on laying performance, egg quality, plasma antioxidant balance, and Se content in eggs of laying Longyan ducks. The authors observed that the basal Se level in the diet (0.15 mg/kg) was sufficient to maintain productive performance and egg quality traits. However, additional supplementation with Se-enriched yeast significantly improved the birds’ antioxidant balance and increased Se deposition in the eggs, resulting in the production of naturally selenium-enriched eggs.

Consistent with these findings, Abbas et al. [90] investigated the effect of supplementation with Se-enriched yeast on mitigating the decline in performance, immunity, and physiological parameters induced by heat stress in laying hens. The authors observed that heat stress compromised all productive aspects of the birds throughout the experiment; however, supplementation with Se-rich yeast exerted a protective effect, improving the performance of both heat-stressed birds and those maintained under thermoneutral conditions.

Beyond the nutritional aspect, this study reinforces the importance of valorizing industrial coproducts. The integration of Se-enriched yeast into DDGS not only adds functionality to an abundant input of the ethanol chain but also provides an economically viable alternative to meet the enriched feed market. Such a strategy can reduce supplementation costs while positioning DDGS as a higher value-added product.

Finally, the results presented here pave the way for new applied research lines, including the optimization of metabolic processes, whether under aerobic or anaerobic conditions, for different production scales, as well as the evaluation of the impact of this enriched DDGS on the zootechnical performance of different animal species.

## Conclusion

The commercialization of Se-enriched yeasts has been ongoing for some time; however, most of the available products contain relatively low Se concentrations (1–2 mg g⁻¹ dry yeast) and, in many cases, in forms with limited bioavailability. In the present study, we demonstrated that *Saccharomyces cerevisiae* (Thermosacc strain), when cultivated in corn hydrolysate supplemented with different Na₂SeO₃ concentrations, was able to accumulate significantly higher Se levels, reaching up to 6.15 mg g⁻¹ dry yeast under aerobic conditions—approximately 50% higher than previously reported in the literature.

Additionally, all aerobic treatments exhibited higher levels of the different Se fractions analyzed (total, organic, and SeMet). Our results also showed that the efficiency of converting inorganic Se into organic forms, particularly SeMet, varied according to the metabolic condition and supplemented concentration, being most pronounced in the treatments with 200 mg L⁻¹. This result suggests the existence of a physiological limit for intracellular Se accumulation, beyond which cellular detoxification mechanisms are activated.

This study demonstrated that different metabolic conditions can enhance the value of yeasts present in DDGS (distillers dried grains with solubles), transforming this coproduct of the ethanol industry into a functional supplement with high Se bioavailability, antioxidant properties, and potential benefits for immune health, reproduction, and stress resistance in animals. Incorporating this strain into DDGS not only reduces supplementation costs but also offers a more efficient and less toxic Se source, since all supplemented treatments showed conversion rates above 50% to organic Se. These findings expand the understanding of Se biotransformation in yeasts and highlight Se-enriched DDGS as a promising and innovative strategy for animal nutrition and potentially the human food chain, with relevant biotechnological and socioeconomic implications.

## Data Availability

No datasets were generated or analysed during the current study.

## References

[1] Joy EJM, Kumssa DB, Broadley MR et al (2015) Dietary mineral supplies in Malawi: spatial and socioeconomic assessment. BMC Nutr 1:42. 10.1186/s40795-015-0036-4

[2] Gashu D, Nalivata PC, Amede T et al (2021) The nutritional quality of cereals varies geospatially in Ethiopia and Malawi. Nature 594:71–76. 10.1038/s41586-021-03559-3 34012114 10.1038/s41586-021-03559-3 PMC8172382

[3] Rayman MP (2004) The use of high-selenium yeast to raise selenium status: how does it measure up? Br J Nutr 92:557–573. 10.1079/bjn20041251 15522125 10.1079/bjn20041251

[4] Gui J-Y, Rao S, Gou Y et al (2022) Comparative study of the effects of selenium yeast and sodium selenite on selenium content and nutrient quality in broccoli florets (*Brassica oleracea* L. var. italica). J Sci Food Agric 102:1707–1718. 10.1002/jsfa.11511 34460116 10.1002/jsfa.11511

[5] Wang H, Yang S, Chen Y et al (2024) Comprehensive distribution and species of selenium in Se-enriched *Pichia kudriavzevii* 1845. Food Chem 438:137966. 10.1016/j.foodchem.2023.137966 37976881 10.1016/j.foodchem.2023.137966

[6] Bansal MP, Kaur T (2002) Growth characteristics and selenium status changes of yeast cells with inorganic and organic selenium supplementation: selenium, a chemopreventive agent. J Med Food 5:85–90. 10.1089/109662002760178168 12487755 10.1089/109662002760178168

[7] Polatajko A, Śliwka-Kaszyńska M, Dernovics M et al (2004) A systematic approach to selenium speciation in selenized yeast. J Anal Spectrom 19:114–120. 10.1039/b308756p

[8] Rayman MP (2000) The importance of selenium to human health. Lancet 356:233–241. 10.1016/S0140-6736(00)02490-9 10963212 10.1016/S0140-6736(00)02490-9

[9] Ogra Y, Shimizu M, Takahashi K, Anan Y (2018) Biotransformation of organic selenium compounds in budding yeast, *Saccharomyces cerevisiae*. Metallomics 10:1257–1263. 10.1039/C8MT00176F 30110033 10.1039/c8mt00176f

[10] Kitajima T, Chiba Y (2013) Selenomethionine metabolism and its toxicity in yeast. Biomol Concepts 4:611–616. 10.1515/bmc-2013-0033 25436761 10.1515/bmc-2013-0033

[11] Kahakachchi C, Boakye HT, Uden PC, Tyson JF (2004) Chromatographic speciation of anionic and neutral selenium compounds in Se-accumulating *Brassica juncea* (Indian mustard) and in selenized yeast. J Chromatogr A 1054:303–312. 10.1016/j.chroma.2004.07.083 15553157

[12] Jach ME, Serefko A, Ziaja M, Kieliszek M (2022) Yeast protein as an easily accessible food source. Metabolites 12(1):63. 10.3390/metabo12010063 35050185 10.3390/metabo12010063 PMC8780597

[13] Böttger C, Südekum KH (2018) Review: protein value of distillers dried grains with solubles (DDGS) in animal nutrition as affected by the ethanol production process. Anim Feed Sci Technol 244:11–17. 10.1016/j.anifeedsci.2018.07.018

[14] Christopher A, Ostrander J, Mathew J et al (2023) Corn distiller’s dried grains with solubles as a target for fermentation to improve bioactive functionality for animal feed and as a source for a novel microorganism with antibacterial activity. Front Food Sci Technol 3:1075789. 10.3389/frfst.2023.1075789

[15] Shad ZM, Venkitasamy C, Wen Z (2021) Corn distillers dried grains with solubles: production, properties, and potential uses. Cereal Chem 98:999–1019. 10.1002/cche.10445

[16] Duarte VD, Valentini MHK, Santos GB et al (2022) Biocombustíveis: Uma revisão sobre o panorama histórico, produção e aplicações do biodiesel. Meio Ambiente (Brasil) 4:050–068. 10.5281/zenodo.7325288

[17] UNICAdata (2025) 2024/2025 Sugarcane Season in Brazil. União da Indústria de Cana-de-Açúcar e Bioenergia (UNICA). https://www.unicadata.com.br. Accessed 01 August 2025

[18] Food and Agriculture Organization of the United Nations (FAO) (2025) FAOSTAT Statistical Database: Crops and livestock products. FAO, Rome. https://www.fao.org/faostat Accessed 01 August 2025

[19] Yoshinaga M, How S, Blanco D et al (2018) Directed evolution of *Saccharomyces cerevisiae* for increased selenium accumulation. Microorganisms 6(3):81. 10.3390/microorganisms6030081 30082639 10.3390/microorganisms6030081 PMC6165298

[20] Lagunas R (1979) Energetic irrelevance of aerobiosis for *S. cerevisiae* growing on sugars. Mol Cell Biochem 27:139–146. 10.1007/BF00215362 390364 10.1007/BF00215362

[21] Sica P, Prado LMLM, Granja P et al (2021) Effects of energy cane (*Saccharum* spp.) juice on corn ethanol (*Zea mays*) fermentation efficiency: integration towards a more sustainable production. Fermentation 7(1):30. 10.3390/fermentation7010030

[22] Douradinho R, Sica P, Tonoli F et al (2023) Osmotic stress alleviation in *Saccharomyces cerevisiae* for high ethanol fermentations with different wort substrates. Stresses 3:813–826. 10.3390/stresses3040055

[23] Mota LA, Silva APM, Salva EA et al (2022) Ability of the *Saccharomyces cerevisiae* Y904 to tolerate and adapt to high concentrations of selenium. Brazilian J Biosystems Eng 16. 10.18011/bioeng.2022.v16.1066

[24] Lim HC, Shin HS (2013) Fed-batch cultures: principles and applications of semi-batch bioreactors. Cambridge University Press, Cambridge

[25] Pierce JS (1970) Institute of brewing: analysis committee measurement of yeast viability. J Inst Brew 76:442–443. 10.1002/j.2050-0416.1970.tb03325.x

[26] Zago E, Silva L, Bernardino CD, Amorim HV (1996) Métodos analíticos Para o controle Da produção de álcool e açúcar. Fermentec, Piracicaba

[27] Aiba S, Shoda M, Nagatani M (1968) Kinetics of product inhibition in alcohol fermentation. Biotechnol Bioeng 10:845–864. 10.1002/bit.260100610 10.1002/(sici)1097-0290(20000320)67:6<671::aid-bit6>3.0.co;2-w 10699849

[28] Calegari RP, da Silva EA, da Silva APM et al (2023) Wort disinfection treatment with electron beam for bioethanol production. Sci Agric 80:e20210260. 10.1590/1678-992X-2021-0260

[29] Catarino S, Trancoso IM, Bruno De Sousa R, Curvelo-Garcia AS (2010) Grape must mineralization by high pressure microwave diges-tion for trace element analysis: development of a procedure. Cienc Tec Vitivinícola 25(2):87–93

[30] Silva EL, Roldan S, Giné MF (2009) Simultaneous preconcentration of copper, zinc, cadmium, and nickel in water samples by cloud point extraction using 4-(2-pyridylazo)-resorcinol and their determination by inductively coupled plasma optic emission spectrometry. J Hazard Mater 171:1133–1138. 10.1016/j.jhazmat.2009.06.127 19646812 10.1016/j.jhazmat.2009.06.127

[31] Alijan S, Hosseini M, Esmaeili S et al (2022) Impact of ultrasound and medium condition on production of selenium-enriched yeast q. Electron J Biotechnol 60:36–42. 10.1016/j.ejbt.2022.08.004

[32] Roepcke CBS, Vandenberghe LPS, Soccol CR (2011) Optimized production of *Pichia guilliermondii* biomass with zinc accumulation by fermentation. Anim Feed Sci Technol 163:33–42. 10.1016/j.anifeedsci.2010.09.018

[33] R Core Team (2024) A language and environment for statistical computing. R Foundation for Statistical Computing, Vienna, Austria. https://www.r-project.org/

[34] Fagan S, Owens R, Ward P et al (2015) Biochemical comparison of commercial selenium yeast preparations. Biol Trace Elem Res 166:245–259. 10.1007/s12011-015-0242-6 25855372 10.1007/s12011-015-0242-6

[35] Jiang Z, Chi J, Li H et al (2021) Effect of chitosan oligosaccharide-conjugated selenium on improving immune function and blocking gastric cancer growth. Eur J Pharmacol 891:173673. 10.1016/j.ejphar.2020.173673 33098836 10.1016/j.ejphar.2020.173673

[36] Mushtaq M, Fatima M, Shah SZH et al (2022) Effects of sodium selenite, selenium methionine, and selenium yeast on growth performance, carcass composition, blood biochemistry, and antioxidant status of intensively reared Hypophthalmichthys molitrix. Aquac Rep 24:101182. 10.1016/j.aqrep.2022.101182

[37] Muhammad AI, Mohamed DAA, Chwen LT et al (2021) Effect of sodium selenite, selenium yeast, and bacterial enriched protein on chicken egg yolk color, antioxidant profiles, and oxidative stability. Foods 10(4):871. 10.3390/foods10040871 33923439 10.3390/foods10040871 PMC8073331

[38] Martiniano SE, Rodrigues Philippini R, Franco-Marcelino PR, da Silva SS (2022) Effect of selenium uptake on growth metabolism in yeasts for the production of enriched single-cell protein using agro-industrial by-products. Biomass Convers Biorefin 12:3975–3983. 10.1007/s13399-020-00885-w

[39] Kieliszek M, Błażejak S, Bzducha-Wróbel A, Kot AM (2019) Effect of selenium on lipid and amino acid metabolism in yeast cells. Biol Trace Elem Res 187:316–327. 10.1007/s12011-018-1342-x 29675568 10.1007/s12011-018-1342-x PMC6315055

[40] Barroso MF, Luna MA, Moyano F et al (2018) Study of lipid peroxidation and ascorbic acid protective role in large unilamellar vesicles from a new electrochemical performance. Bioelectrochemistry 120:120–126. 10.1016/j.bioelechem.2017.12.003 29247891 10.1016/j.bioelechem.2017.12.003

[41] Luo J, Li X, Li X et al (2018) Selenium-rich yeast protects against aluminum-induced peroxidation of lipide and inflammation in mice liver. BioMetals 31:1051–1059. 10.1007/s10534-018-0150-2 30288658 10.1007/s10534-018-0150-2

[42] Mota LA, Calegari RP, Pinto AU et al (2025) Selenium-biofortified spent yeast cultivated in corn hydrolysate: antioxidant response and biomass production under aerobic and anaerobic conditions. Int Microbiol. 10.1007/s10123-025-00722-y 41087792 10.1007/s10123-025-00722-y PMC12727741

[43] Sica P, Tonoli F, Silverio MS et al (2024) Pre-adaptation of yeast (*Saccharomyces cerevisiae*) strains to very high gravity can improve fermentation parameters and reduce osmotic stress. Biomass Convers Biorefin 15:9123–9137. 10.1007/s13399-024-05746-4

[44] Faramarzi S, Anzabi Y, Jafarizadeh-Malmiri H (2020) Nanobiotechnology approach in intracellular selenium nanoparticle synthesis using *Saccharomyces cerevisiae*—fabrication and characterization. Arch Microbiol 202:1203–1209. 10.1007/s00203-020-01831-0 32077990 10.1007/s00203-020-01831-0

[45] Wang L, Zhao XQ, Xue C, Bai FW (2013) Impact of osmotic stress and ethanol inhibition in yeast cells on process oscillation associated with continuous very-high-gravity ethanol fermentation. Biotechnol Biofuels 6(1):133. 10.1186/1754-6834-6-133 24041271 10.1186/1754-6834-6-133 PMC3849797

[46] Kieliszek M, Bierla K, Jiménez-Lamana J et al (2020) Metabolic response of the yeast *Candida utilis* during enrichment in selenium. Int J Mol Sci 21:55287. 10.3390/ijms21155287 10.3390/ijms21155287 PMC7432028 32722488

[47] Herrero E, Wellinger RE (2015) Yeast as a model system to study metabolic impact of selenium compounds. Microb Cell 2:139–149. 10.15698/mic2015.05.200 28357286 10.15698/mic2015.05.200 PMC5349236

[48] Nie X, Yang X, He J et al (2023) Bioconversion of inorganic selenium to less toxic selenium forms by microbes: a review. Front Bioeng Biotechnol 11:1167123. 10.3389/fbioe.2023.1167123 36994362 10.3389/fbioe.2023.1167123 PMC10042385

[49] Bodnar M, Szczyglowska M, Konieczka P, Namiesnik J (2016) Methods of selenium supplementation: bioavailability and determination of selenium compounds. Crit Rev Food Sci Nutr 56:36–55. 10.1080/10408398.2012.709550 24987868 10.1080/10408398.2012.709550

[50] Grant CM (2001) Role of the glutathione/glutaredoxin and thioredoxin systems in yeast growth and response to stress conditions. Mol Microbiol 39:533–541. 10.1046/j.1365-2958.2001.02283.x 11169096 10.1046/j.1365-2958.2001.02283.x

[51] Hachemi MA, Cardoso D, De Marco M et al (2023) Inorganic and organic selenium speciation of seleno-yeasts used as feed additives: new insights from elemental selenium determination. Biol Trace Elem Res 201:5839–5847. 10.1007/s12011-023-03633-z 36934195 10.1007/s12011-023-03633-z PMC10620252

[52] Mapelli V, Hillestrøm PR, Patil K et al (2012) The interplay between sulphur and selenium metabolism influences the intracellular redox balance in *Saccharomyces cerevisiae*. FEMS Yeast Res 12:20–32. 10.1111/j.1567-1364.2011.00757.x 22093810 10.1111/j.1567-1364.2011.00757.x

[53] Kieliszek M, Błazejak S (2018) Speciation analysis of selenium in *Candida utilis* yeast cells using HPLC-ICP-MS and UHPLC-ESI-Orbitrap MS techniques. Appl Sci 8(11):2050. 10.3390/app8112050

[54] Bierła K, Suzuki N, Ogra Y et al (2017) Identification and determination of selenohomolanthionine – the major selenium compound in Torula yeast. Food Chem 237:1196–1201. 10.1016/j.foodchem.2017.06.042 28763969 10.1016/j.foodchem.2017.06.042

[55] Esmaeili S, Khosravi-Darani K, Pourahmad R, Komeili R (2012) An experimental design for production of selenium-enriched yeast. World Appl Sci J 19(1):31–37. 10.5829/IDOSI.WASJ.2012.19.01.2634

[56] Rampler E, Rose S, Wieder D et al (2012) Monitoring the production process of selenized yeast by elemental speciation analysis. Metallomics 4:1176–1184. 10.1039/c2mt20138k 23072765 10.1039/c2mt20138k

[57] Hu T, Hui G, Li H, Guo Y (2020) Selenium biofortification in *Hericium erinaceus* (Lion’s Mane mushroom) and its in vitro bioaccessibility. Food Chem 331:127287. 10.1016/j.foodchem.2020.127287 32563801 10.1016/j.foodchem.2020.127287

[58] Kieliszek M, Błażejak S, Gientka I, Bzducha-Wróbel A (2015) Accumulation and metabolism of selenium by yeast cells. Appl Microbiol Biotechnol 99:5373–5382. 10.1007/s00253-015-6650-x 26003453 10.1007/s00253-015-6650-x PMC4464373

[59] Kordialik-Bogacka E (2011) Surface properties of yeast cells during heavy metal biosorption. Cent Eur J Chem 9:348–351. 10.2478/s11532-011-0008-8

[60] Klis FM, Boorsma A, De Groot PWJ (2006) Cell wall construction in Saccharomyces cerevisiae. Yeast 23:185–202. 10.1002/yea.1349 16498706 10.1002/yea.1349

[61] Lipke PN, Ovalle R (1998) Cell wall architecture in yeast: new structure and new challenges. J Bacteriol 180:3735–3740. 10.1128/jb.180.15.3735-3740.1998 9683465 10.1128/jb.180.15.3735-3740.1998 PMC107352

[62] Gong A, Liu W, Lin Y et al (2023) Adaptive laboratory evolution reveals the selenium efflux process to improve selenium tolerance mediated by the membrane sulfite pump in *Saccharomyces cerevisiae*. Microbiol Spectr 11:e01326-23. 10.1128/spectrum.01326-23 37098949 10.1128/spectrum.01326-23 PMC10269739

[63] McDermott JR, Rosen BP, Liu Z (2010) Jen1p: a high affinity selenite transporter in yeast. Mol Biol Cell 21:3934–3941. 10.1091/mbc.E10 20861301 10.1091/mbc.E10-06-0513 PMC2982120

[64] Gharieb MM, Gadd GM (2004) The kinetics of 75[Se]-selenite uptake by *Saccharomyces cerevisiae* and the vacuolization response to high concentrations. Mycol Res. 10.1017/S0953756204001418 15757177 10.1017/s0953756204001418

[65] Suhajda A, Hegóczki J, Janzsó B et al (2000) Preparation of selenium yeasts I. preparation of selenium-enriched *Saccharomyces cerevisiae*. J Trace Elem Med Biol 14:43–47 10836533 10.1016/S0946-672X(00)80022-X

[66] Golubev VL, Golubev NV (2002) Selenium tolerance of yeasts. microbiology (N Y) 71:455–459. 10.1023/A:1019829107239 12244713

[67] Asker MMS, Ibrahim GS, Shaltout AA, Mahmoud MG (2020) Accumulation of selenium by different yeast cells. Plant Arch 20(Suppl 1):1465–1468

[68] Zhang GC, Wang DH, Wang DH, Wei GY (2017) The mechanism of improved intracellular organic selenium and glutathione contents in selenium-enriched *Candida utilis* by acid stress. Appl Microbiol Biotechnol 101:2131–2141. 10.1007/s00253-016-8016-4 27896382 10.1007/s00253-016-8016-4

[69] da Silva APM, Sica P, Pires L, de AN et al (2023) Integration of corn and cane for ethanol production: effects of *Lactobacilli* contamination on fermentative parameters and use of ionizing radiation treatment for disinfection. Fermentation 9(2):89. 10.3390/fermentation9020089

[70] Nasim MJ, Zuraik MM, Abdin AY et al (2021) Selenomethionine: a pink trojan redox horse with implications in aging and various age-related diseases. Antioxidants 10:1375. 10.3390/antiox10060882 34072794 10.3390/antiox10060882 PMC8229699

[71] Rao Y, McCooeye M, Windust A et al (2010) Mapping of selenium metabolic pathway in yeast by liquid chromatography-orbitrap mass spectrometry. Anal Chem 82:8121–8130. 10.1021/ac1011798 20825195 10.1021/ac1011798

[72] EFSA (2012) Scientific opinion on safety and efficacy of selenium in the form of organic compounds produced by the selenium-enriched yeast *Saccharomyces cerevisiae* NCYC R646 (Selemax 1000/2000) as feed additive for all species. EFSA J 10(7):2778. 10.2903/j.efsa.2012.2778 42016097 10.2903/j.efsa.2012.2778 PMC13093067

[73] Wu J, Hong L, Shi M (2021) Production of methylselenocysteine in *Saccharomyces cerevisiae* LG6 by continuous fermentation. Bioresour Technol Rep 13:100627. 10.1016/j.biteb.2021.100627

[74] Ward P, Chadha M, Connolly C et al (2019) A comparative assessment of water-soluble selenium metabolites in commercial selenised yeast supplements by liquid chromatography-electrospray ionisation QTOF-MS. Int J Mass Spectrom 439:42–52. 10.1016/j.ijms.2018.10.040

[75] Sun H, Zhao L, Xu ZJ et al (2021) Hydroxy-selenomethionine improves the selenium status and helps to maintain broiler performances under a high stocking density and heat stress conditions through a better redox and immune response. Antioxidants 10(10):1542. 10.3390/antiox10101542 34679677 10.3390/antiox10101542 PMC8532863

[76] Briviba K, Roussyn I, Sharov VS, Sies H (1996) Attenuation of oxidation and nitration reactions of peroxynitrite by selenomethionine, Selenocystine and Ebselen. Biochem J 319(1):13–15. 10.1042/bj3190013 8870642 10.1042/bj3190013 PMC1217728

[77] Cui X, Wang Z, Tan Y et al (2021) Selenium yeast dietary supplement affects rumen bacterial population dynamics and fermentation parameters of Tibetan sheep (*Ovis aries*) in alpine meadow. Front Microbiol 12:663945. 10.3389/fmicb.2021.663945 34276597 10.3389/fmicb.2021.663945 PMC8283570

[78] Bano I, Malhi M, Khatri P et al (2019) Effect of dietary selenium yeast supplementation on morphology and antioxidant status in testes of young goat. Pak J Zool 51:979–988. 10.17582/journal.pjz/2019.51.3.979.988

[79] Huang Q, Wang S, Yang X et al (2023) Effects of organic and inorganic selenium on selenium bioavailability, growth performance, antioxidant status and meat quality of a local beef cattle in China. Front Vet Sci. 10.3389/fvets.2023.1171751 37180071 10.3389/fvets.2023.1171751 PMC10172650

[80] Yu D, Liu Y, Wan J et al (2025) Effects of different forms of organic selenium on growth performance, antioxidant capacity, and intestinal health in rice field eel (*Monopterus albus*). Animals 15(13):1949. 10.3390/ani15131949 40646848 10.3390/ani15131949 PMC12249458

[81] Ouerdane L, Mester Z (2008) Production and characterization of fully selenomethionine-labeled *Saccharomyces cerevisiae*. J Agric Food Chem 56:11792–11799. 10.1021/jf8018479 19035646 10.1021/jf8018479

[82] Tarze A, Dauplais M, Grigoras I et al (2007) Extracellular production of hydrogen selenide accounts for thiol-assisted toxicity of selenite against *Saccharomyces cerevisiae*. J Biol Chem 282:8759–8767. 10.1074/jbc.M610078200 17261587 10.1074/jbc.M610078200

[83] Garbisu C, Ishii T, Leighton T, Buchanan BB (1996) Bacterial reduction of selenite to elemental selenium. Chem Geol 132:199–204. 10.1016/S0009-2541(96)00056-3

[84] Malkowski MG, Quartley E, Friedman AE et al (2007) Blocking S-adenosylmethionine synthesis in yeast allows selenomethionine incorporation and multiwavelength anomalous dispersion phasing. Proc Nat Acad Sci U S A 104:14523–14528. 10.1073/pnas.061033710 10.1073/pnas.0610337104 PMC1850019 17426150

[85] Kitajima T, Jigami Y, Chiba Y (2012) Cytotoxic mechanism of selenomethionine in yeast. J Biol Chem 287(13):10032–10038. 10.1074/jbc.M111.324244 22311978 10.1074/jbc.M111.324244 PMC3323055

[86] Wiebe MG, Rintala E, Tamminen A et al (2008) Central carbon metabolism of Saccharomyces cerevisiae in anaerobic, oxygen-limited and fully aerobic steady-state conditions and following a shift to anaerobic conditions. FEMS Yeast Res 8:140–154. 10.1111/j.1567-1364.2007.00234.x 17425669 10.1111/j.1567-1364.2007.00234.x

[87] Wickramasuriya SS, Park I, Lee Y, Lillehoj HS (2023) Effect of dietary organic selenium on growth performance, gut health, and coccidiosis response in broiler chickens. Animals 13(9):1560. 10.3390/ani13091560 37174598 10.3390/ani13091560 PMC10177327

[88] Islam Z, Ikram M, Naz S et al (2024) Effect of selenium-enriched yeast diet on performance, biochemistry, and selenium concentration in meat and egg contents of laying Japanese quails. Arch Anim Breed 67:493–504. 10.5194/aab-67-493-2024 10.5194/aab-67-493-2024 PMC13207711 42205497

[89] Zhang X, Tian L, Zhai S et al (2020) Effects of selenium-enriched yeast on performance, egg quality, antioxidant balance, and egg selenium content in laying ducks. Front Vet Sci 7:591. 10.3389/fvets.2020.00591 33102547 10.3389/fvets.2020.00591 PMC7500446

[90] Abbas AO, Alaqil AA, Mehaisen GMK, El Sabry MI (2022) Effect of organic selenium-enriched yeast on relieving the deterioration of layer performance, immune function, and physiological indicators induced by heat stress. Front Vet Sci. 10.3389/fvets.2022.880790 35573399 10.3389/fvets.2022.880790 PMC9096893

